# Three-day regimen of oseltamivir for post-exposure prophylaxis of influenza in hospital wards: a study protocol for a prospective, multi-center, single-arm trial

**DOI:** 10.1186/s12879-021-06602-w

**Published:** 2021-08-30

**Authors:** Nobuhisa Ishiguro, Yoichi M. Ito, Sumio Iwasaki, Miki Nagao, Hideki Kawamura, Shinichiro Kanai, Yoko Nukui, Koichi Tokuda, Takayuki Miyara, Hidetoshi Igari, Koichi Yamada, Hiroki Chikumi, Chiaki Sano, Ryuji Koike, Tetsuya Yagi, Nobuo Murakami, Japan Infection Prevention, Control Conference for National, Public University Hospitals

**Affiliations:** 1 Division of Infection Control, Hokkaido University Hospital, Kita 14, Nishi 5, Kita-Ku, Sapporo, Hokkaido 060-8648 Japan; 2 Department of Statistical Data Science, The Institute of Statistical Mathematics, 10-3 Midori-cho, Tachikawa, Tokyo 190-8562 Japan; 3 Department of Infection Control and Prevention, Kyoto University Hospital, 54 Kawahara-cho, Shogoin, Sakyo-ku, Kyoto, Kyoto 606-8507 Japan; 4 Department of Infection Control and Prevention, Division of Medical and Environmental Safety, Kagoshima University Hospital, 8-35-1 Sakuragaoka, Kagoshima, Kagoshima 890-8544 Japan; 5 Division of Infection Control, Shinshu University Hospital, 3-1-1 Asahi, Matsumoto, Nagano 390-8621 Japan; 6 Department of Infection Control and Prevention, Medical Hospital, Tokyo Medical and Dental University, 1-5-45 Yushima, Bunkyo-ku, Tokyo, 113-8510 Japan; 7 Department of Infection Control and Laboratory Diagnostics, Tohoku University Graduate School of Medicine, 2-1 Seiryo-machi, Aoba-ku, Sendai, Miyagi 980-8575 Japan; 8 Infection Control Team, Kobe University Hospital, 7-5-2 Kusunoki-cho, Chuo-ku, Kobe, Hyogo 650-0017 Japan; 9 Division of Infection Control, Chiba University Hospital, 1-8-1 Inohana, Chuo-ku, Chiba, Chiba 260-8677 Japan; 10 Department of Infection Control Science, Graduate School of Medicine, Osaka City University, 1-4-3 Asahimachi, Abeno-ku, Osaka, Osaka 545-8585 Japan; 11 Center for Infectious Diseases, Tottori University Hospital, 36-1 Nishi-cho, Yonago, Tottori 683-8504 Japan; 12 Division of Infection Control, Shimane University Hospital, 89-1 Enya-cho, Izumo, Shimane 693-8501 Japan; 13 Department of Infectious Diseases, Nagoya University Hospital, 65 Tsurumai-cho, Showa-ku, Nagoya, Nagoya 466-8560 Japan; 14 Center for Regional Medicine, Gifu University School of Medicine, 1-1 Yanagido, Gifu, Gifu 501-1194 Japan

**Keywords:** Oseltamivir, Post-exposure prophylaxis, Influenza, Hospital wards

## Abstract

**Background:**

In a previous retrospective observational study, a 3-day regimen of oseltamivir as post-exposure prophylaxis (PEP) for preventing transmission of influenza in wards was shown to be comparable to 7- to 10-day regimens provided index cases were immediately separated from close contacts. In order to confirm the efficacy of a 3-day regimen, we started to conduct a prospective, multi-center, single-arm trial.

**Methods:**

This study is a prospective, multi-center, single-arm study designed by the Sectional Meeting of Clinical Study, Japan Infection Prevention and Control Conference for National and Public University Hospitals. Index patients with influenza are prescribed a neuraminidase inhibitor and are discharged immediately or transferred to isolation rooms. The close contacts are given oseltamivir as 75 mg capsules once daily for adults or 2 mg/kg (maximum of 75 mg) once daily for children for 3 days as PEP. All close contacts are monitored for development of influenza for 7 days after starting PEP.

**Discussion:**

A 3-day regimen of oseltamivir as PEP has advantages over 7- to 10-day regimens in terms of costs, medication adherence and adverse effects.

*Trial registration* The Institutional Review Board of Hokkaido University Hospital for Clinical Research, 015-0518, registered on November 11, 2016. UMIN Clinical Trials Registry, UMIN000024458, disclosed on October 31, 2016. https://upload.umin.ac.jp/cgi-open-bin/ctr_e/ctr_view.cgi?recptno=R000027881. Japan Registry of Clinical Trials, jRCTs011180015, disclosed on March 14, 2019. https://jrct.niph.go.jp/latest-detail/jRCTs011180015

## Background

Post-exposure prophylaxis (PEP) using oseltamivir has been shown to be effective for reducing secondary infection of influenza in hospital wards [1]. A retrospective observational study showed that a 3-day regimen of oseltamivir as PEP for preventing transmission of influenza in wards is comparable to 7- to 10-day regimens provided index cases are immediately separated from close contacts [2]. In order to confirm the efficacy of a 3-day regimen of oseltamivir as PEP, we started to conduct a prospective, multi-center, single-arm trial.

## Methods/design

### Aim

The objective of this study is to investigate the efficacy of a 3-day regimen of oseltamivir for PEP of influenza in hospital wards.

### Study setting

This study is a prospective, multi-center, single-arm study designed by the Sectional Meeting of Clinical Study, Japan Infection Prevention and Control Conference for National and Public University Hospitals in Japan. This trial will involve patients from Hokkaido University Hospital, Kagoshima University Hospital, Shinshu University Hospital, Tokyo Medical and Dental University Hospital, Tohoku University Hospital, Kobe University Hospital, Chiba University Hospital, Osaka City University Hospital, Tottori University Hospital and Shimane University Hospital in Japan.

### Endpoints

The primary endpoint is the incidence of influenza for 7 days after starting PEP using oseltamivir by use of a 3-day regimen. The development of influenza is defined as development of influenza-like symptoms (e.g., fever, cough and runny nose) with a positive immunochromatographic test (ICT). The secondary endpoints are the associations of the incidence of influenza for 7 days after starting PEP using oseltamivir by use of a 3-day regimen with age, sex, influenza vaccination and underlying conditions of the patients because of the possibility of development of influenza in elderly patients, unvaccinated patients and immunocompromised patients even if they are prescribed oseltamivir.

### Study design

The index patients with influenza are prescribed a neuraminidase inhibitor and are isolated from close contacts [2]. Close contacts who fulfill all of the criteria described in “Eligibility and exclusion” are given oseltamivir [75 mg capsules once daily for adults or 2 mg/kg (maximum of 75 mg) once daily for children] for 3 days as PEP. Since PEP with anti-influenza agents is widely practiced in Japan for both healthcare workers and patients, even for those who have already received vaccination, it is difficult to conduct a randomized controlled trial with comparison of a 3-day regimen of oseltamivir and a placebo control. The results of previous studies are destined to be used as historical controls [1–4].

### Eligibility and exclusion

Eligible participants are (1) patients over 1 year of age, (2) patients sharing a room with index patients with influenza who are separated immediately (ex. by discharge or transferring to isolation rooms), (3) patients in whom administration of oseltamivir as PEP is started within 48 h after onset of fever of the index patients and (4) patients for whom written informed consent to participate in the study is obtained by the physicians participating in this study, from the patients themselves or from proxies. The following patients are not eligible for participation in this study: (1) patients with impairment of renal function (creatinine clearance ≤ 30 ml/min) and (2) patients between 10 and 19 years of age, because the Japanese Ministry of Health, Labor and Welfare issued a warning against the use of oseltamivir in teenagers, except for those at high risks, after reports of two suicides by teenagers shortly after ingestion of oseltamivir in March 2007 [5].

### Study intervention and timeline of the study

The following information will be obtained from each patient’s record at the time of enrolment: (1) body temperature, respiratory symptoms (e.g., cough and runny nose) and results of an immunochromatographic test (ICT) of influenza in index patients, (2) clinical history including age, sex, body weight, influenza vaccination, past infection with influenza, underlying disease and medical history, and results of laboratory tests including hemoglobin, white blood count, aspartate aminotransferase (AST), alanine aminotransferase (ALT) and creatinine for the close contacts. Eligible participants for whom administration of oseltamivir is started as PEP are monitored for influenza-like symptoms (fever and respiratory symptoms) for 7 days (Table 1). If the eligible patients have influenza-like symptoms with a positive immunochromatographic test (ICT), they are regarded as having developed influenza. Information on adverse events will be obtained regardless of their grade of severity. This trial was declared and registered on November 11, 2016 (see “Trial status”).

**Table 1:** Schedule of data collection

	Before PEP^a^	Observation period						
		Day 1	Day 2	Day 3	Day 4	Day 5	Day 6	Day 7
Informed consent	X							
Clinical assessment^b^	X							
Laboratory test^c^	X							
PEP^a^		X	X	X				
Fever		X	X	X	X	X	X	X
Respiratory symptoms^d^		X	X	X	X	X	X	X
Immunochromatographic testing^e^		X	X	X	X	X	X	X
Adverse events		X	X	X	X	X	X	X

^a^Post-exposure prophylaxis

^b^Clinical assessment: history including age, sex, body weight, influenza vaccination, past infection with influenza, underlying disease and medical history

^c^Laboratory test: hemoglobin, white blood count, aspartate aminotransferase (AST), alanine aminotransferase (ALT) and creatinine

^d^Respiratory symptoms: cough or sneezing

^e^If the close contacts with index patients with influenza have fever or respiratory symptoms within 7 days after PEP, immunochromatographic testing of influenza will be carried out

### Sample size

In our previous study [2], we prophylactically administered oseltamivir for 3 days to 212 patients, and only 2 (0.9%) of the patients developed influenza. In preceding clinical trials [1, 3, 4], the proportion of patients with influenza infection in the placebo group was about 7%. In this study, it is assumed that the proportion of patients with influenza will be lower than that in preceding studies due to the different study environments. Based on the idea of non-inferiority margin [6], the clinically meaningful margin is set to be 50% of pre-existing risk difference (δ = 7 − 1 = 6%). Therefore, the non-inferiority margin of our study is set to be 3%. From the above argument, we assumed that the proportions of patients with influenza infection are 1% in this study (3-day regimen) and 4% in the null reference. Since the incidence of influenza in a hospital is relatively low and it is possible to confirm the presence or absence of influenza in a short period of time, a binomial distribution was assumed for the incidence. Using the binominal exact test statistic, the expected sample size was calculated to be 253 for detection of a 3% risk difference to achieve a power of 80% at a one-sided significance level of 2.5%. Considering the dropout rate as 10%, the final sample size became 279.

### Statistical analysis

For primary outcome assessment, a binomial (exact) test is performed to test the risk difference of 3% (proportions of patients with influenza infection being 4% for the null reference and 1% for the proposed regimen) at the one-sided significance level of 2.5%. Descriptive statistics are calculated for the secondary outcomes. All analyses are performed using SAS Version 9.4 (Cary, NC: SAS Institute Inc).

### Ethics

The trial will be conducted under ICH E6 (R2) and Ethical Guidelines for Medical and Health Research Involving Human Subjects (Japan). The trial will adhere to the Clinical Trials Act (Japan) from April 1, 2019. Recruitment of participants can start following approval by the IRB of each hospital. The recruitment in Shinshu University Hospital and Kobe University Hospital will be discontinued from April 1, 2019 onward.

### Data quality assurance

The investigators will be responsible for implementing and maintaining quality assurance and quality control systems in accordance with written standard operating procedures. Centralized monitoring will be implemented in accordance with the manual prepared prior to the beginning of the trial.

### Data security

Confidential patient information is kept secure by the rules of medical confidentiality and is treated according to the Act on the Protection of Personal Information in Japan.

### Safety

Participants will be free to withdraw at any time from participation. An investigator may decide to terminate participation in the study if a participant meets an exclusion criterion (either newly developed or previously recognized) that precludes further participation.

### Adverse event (AE)

An adverse event (AE) will be managed according to the FDA’s guidance [7]. All severe adverse event (SAE) information will be reported to the IRB and must be shared with all investigators participating in the study.

## Discussion

The effectiveness of PEP using oseltamivir by use of 7 to 10-day regimens has been shown by several studies [1, 3, 4, 8]. In a previous retrospective observational study conducted from the 2005/06 to 2014/05 influenza seasons, a 3-day regimen of oseltamivir as PEP for preventing transmission of influenza in wards was shown to be comparable to 7- to 10-day regimens of oseltamivir provided index cases were immediately separated from close contacts [2]. A 3-day regimen of oseltamivir as PEP has advantages over 7- to 10-day regimens as regards costs, medication adherence and adverse effects [9].

The incidence rate of influenza within a hospital and the expected incidence rate for the proposed regimen are both low; thus, for the detection of a small difference, the required sample size would become large to achieve nominal statistical power. In order to confirm the efficacy of a 3-day regimen of oseltamivir for PEP of influenza in wards, we started to conduct a prospective, multi-center, single-arm trial of a 3-day regimen of oseltamivir for PEP.

### Trial status

This trial was declared and registered on November 11, 2016. Recruitment into the trial started on November 11, 2016 and will end in March 2024 or until a total of 279 participants have been recruited. Between November 2016 and May 2020, 68 patients were enrolled. Among them, 3 patients developed influenza after starting PEP using oseltamivir by use of a 3-day regimen.

## Data Availability

Not applicable.

## Abbreviations

PEP: Post-exposure prophylaxis
ICH: International Conference on Harmonisation
AE: Adverse event
SAE: Serious adverse event
